# A Novel Structural Vibration Sensing Approach Based on a Miniaturized Inertial Measurement Unit

**DOI:** 10.3390/s25133958

**Published:** 2025-06-25

**Authors:** Liyuan Yu, Zhilei Qiao, Shichao Xing, Yipeng Wu, Hongli Ji

**Affiliations:** 1 State Key Laboratory of Mechanics and Control for Aerospace Structures, Nanjing University of Aeronautics and Astronautics, Nanjing 210016, China; yuliyuan@nuaa.edu.cn (L.Y.); jihongli@nuaa.edu.cn (H.J.); 2 Anhui Joint Key Laboratory of Critical Technologies for High-End Copper-Based New Materials, Tongling University, Tongling 244061, China; 3 Wan Jiang New Industry Technology Development Center, Tongling 244000, China

**Keywords:** inertial measurement unit, structural vibration, vibration sensor, autonomous sensor, Kalman filter

## Abstract

Active or semi-active vibration control systems require real-time vibration information from controlled structures as feedback. However, integrating vibration sensors into some controlled structures remains a challenge due to factors such as mass and signal lines. This issue is particularly prominent in attachment structures located far from the spacecraft, such as robotic arms and solar panels. This paper presents a miniaturized autonomous inertial sensor that can be easily attached to the controlled structure to acquire vibration data and wirelessly transmit the data. We also establish the relationship between cantilevered structural vibration and the inertial acceleration or angular velocity directly measured by the sensor. Consequently, the feedback information for the control system can be calculated by the processor in real-time. This autonomous inertial sensor consists of an inertial measurement unit (IMU) named BMI088 and a common wireless communication unit. An improved Extended Kalman Filter (EKF) algorithm is employed to enhance the quality of the sensing data in practical environments. The experimental results validated the theoretical model, indicating that the miniaturized inertial sensor effectively captures the bending vibration characteristics of the controlled structure.

## 1. Introduction

Vibration widely exists in various mechanical structures and may cause some unwanted results, for instance, reducing the fatigue life of structures, comfort, or safety performance of structural systems, and so on [1]. With the development of modern engineering equipment towards lightweight and intelligent directions, higher requirements for vibration measurement and control have been proposed in fields such as aerospace [2,3,4], robotics [5], and automotive engineering [6]. However, in practical applications, integrating high-performance vibration sensors into complex structures still faces numerous challenges. Traditional sensor solutions based on capacitive or piezoelectric principles struggle to operate in harsh conditions, such as strong magnetic fields, and their single-point measurement characteristics also limit the realization of the distributed vibration detection [7,8]. For attached structures with complex shapes and dynamic characteristics, such as robotic arms and solar sails, the placement of sensors may even lead to issues of system modal interference.

The IMU has attracted widespread attention due to its unique combination of measurement dimensions in the existing sensor technology system. It has been extensively applied across numerous domains, including inertial navigation, smartphones, robotics, unmanned aerial vehicles, automotive, and structural health monitoring [9,10]. The six-axis IMU, capable of simultaneously acquiring three-axis acceleration and three-axis angular velocity parameters, provides comprehensive structural motion data encompassing bending vibrations. Its high sensitivity and resolution make it an ideal choice for precise vibration measurements, particularly in space-constrained and weight-sensitive applications. Currently, low-powered microelectronic devices have become increasingly common in environmental monitoring, structural health monitoring, industrial manufacturing, and other fields [11]. To ensure a small size, low power consumption, low cost, high precision, and high stability, Micro-Electro-Mechanical Systems (MEMSs)-based IMUs have become the preferred solution for inertial sensing applications across multiple industries, thanks to their advantages in miniaturization design [12,13].

S. Sukkarieh et al. [14] proposed a low-cost, redundant, strapdown IMU composed of four low-cost accelerometers and four gyroscopes. The experiment showed that, with Global Positioning System (GPS) assistance, it could estimate the attitude of the aircraft, but without a GPS, it would drift over time. He et al. [15] selected two sets of orthogonal laser gyroscopes as inertial measurement sensors, employing an inertial matching method that effectively measures hull deformation. This approach offers a lower cost and simpler installation compared to GPSs. To meet the higher requirements for size and weight in certain scenarios, Fabian Höflinger et al. [16] developed a wireless micro-IMU that wirelessly transmits data from three-axis accelerometers, three-axis gyroscopes, and three-axis magnetometers to a base station, further reducing the size and weight. Pieter Try and Marion Gebhard [17] designed a sensor device that combines a six-axis IMU with an additional beam structure. Experimental results showed this configuration achieved a 6.2 times higher vibration amplitude and a 480% increased signal energy compared to beamless IMUs, making it more suitable for small vibration measurements. Patrizi et al. [18] studied the performance of an MEMS-IMU under dynamic and random vibration conditions, designing a specialized device to ensure controlled motion and evaluate the IMU’s performance under harsh conditions. In terms of low power consumption, Peihui Yan et al. [19] proposed a real-time high-precision integrated navigation algorithm with dynamic power adaptive adjustment capabilities for a multi-sensor combination of a Global Navigation Satellite System (GNSS), an MEMS-IMU, and odometers. In real-time vehicle-mounted experiments, the Root Mean Square (RMS) statistical value of the overall positioning error in the entire road section was 0.312 m, and the average power consumption was only 141 mW. 

IMUs suffer from a time drift phenomenon [20,21], where measurement errors accumulate over time. To address this issue, the Kalman Filter (KF), as a recursive estimation algorithm, has been widely adopted. This method integrates predicted values and observed values based on the statistical optimal estimation theory [22], effectively suppressing inherent sensor errors and environmental noise. In fields such as positioning, navigation, and sensor networks, the KF employs a real-time data correction mechanism, ensuring the system’s timeliness while being particularly suitable for processing dynamic data from compact sensors under complex conditions like vibrations. To overcome the limitations of the traditional KF in nonlinear system modeling and robustness, researchers have developed variants including the EKF, Robust Kalman Filter (RKF), Unscented Kalman Filter (UKF), Cubature Kalman Filter (CKF), and Probabilistic Kalman Filter (PKF) [23]. Recent studies have also explored novel approaches that integrate KFs with neural networks and machine learning techniques [24,25,26].

This paper addresses the vibration monitoring requirements of beams and similar structures, proposing a vibration sensing method based on a miniature six-axis IMU and an improved EKF. By establishing the relationship between the vibration characteristics of cantilever beam structures and the inertial acceleration and angular velocity of sensors, we developed a miniaturized autonomous inertial sensor that can be easily attached to controlled structural surfaces. This sensor can acquire vibration data and wirelessly transmit the data. In the algorithm design, an improved EKF algorithm with a state variable correction for the accelerometer is adopted, effectively eliminating the dynamic interference from the accelerometer in the vibration environment and enhancing the real-time processing capability and anti-interference performance of the sensor data. Through simulation modeling and the experimental validation, it is confirmed that the method can accurately extract structural bending vibration characteristics, providing a lightweight and high-precision solution for real-time vibration monitoring. This paper elaborates on the vibration sensing mechanism of the six-axis IMU, the challenges of measuring the bending vibration, and the filter algorithm improvement strategy. Additionally, experimental evaluations of the measurement accuracy and real-time performance are provided, offering new research ideas for the development of inertial sensor technologies in structural health monitoring.

The remainder of this paper is organized as follows: Section 2 introduces the hardware and software system of the miniaturized inertial sensor. Section 3 discusses the relationship between the tilt angle and deflection during the cantilever beam bending vibration. Section 4 introduces the improved EKF. Section 5 presents simulation and experimental results, evaluating the measurement accuracy and real-time performance of the proposed new structural vibration sensing method. Section 6 presents the conclusions.

## 2. Miniature Vibration Monitoring System

**Figure 1:**
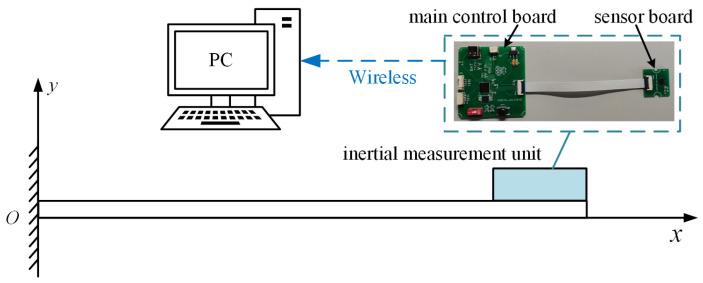
The overall architecture of the miniature inertial measurement unit.

The system is designed to address the bending vibration monitoring requirements of beam structures. The hardware is designed with a split structure, where the sensor board is connected to the main control board via Flexible Printed Circuit (FPC) cables (Jushuo Electronics, Shenzhen, China) and communicates through a Serial Peripheral Interface (SPI) to achieve miniaturization and portability. The sensor board enhances rigidity and seismic performance by reducing the size of the Printed Circuit Board (PCB) substrate (JLC Technology Group Co., Ltd, Shenzhen, China). It utilizes a high-precision six-axis IMU to capture subtle bending vibrations accurately. The main control board employs a highly integrated chip with a floating-point unit (FPU) to improve the floating-point computation speed and incorporates vibration-damping measures to ensure reliability within prolonged vibration environments. The system is structured in a three-tier architecture: the IMU collects vibration data in real-time, which is then transmitted via the SPI to the main control board for the real-time calculation of the tilt angle and amplitude, and finally, the results are wirelessly transmitted to the upper computer. The overall architecture of the miniature IMU is illustrated in Figure 1.

The six-axis IMU utilizes the BMI088 sensor supplied by Bosch Sensortec GmbH (Reutlingen, Germany). It is a MEMS-based IMU that integrates a three-axis 16-bit gyroscope and a three-axis 16-bit accelerometer. Its built-in data processing circuitry converts analog signals into digital data, which is then stored in a register, eliminating the need for external analog-to-digital conversion circuits during the design phase. This significantly reduces the space required for the sensor component and increases the overall integration of the device. As an IMU designed for harsh vibration environments, it also incorporates a hardware low-pass filtering system that effectively filters out high-frequency noise caused by the PCB resonance. The specific parameters of the BMI088 are shown in Table 1.

The main control board is required to perform tasks including reading sensor data, running algorithms to obtain vibration information such as the tilt angle and amplitude, and sending results to the upper computer. The STM32F401CBU6 microcontroller (STMicroelectronics, Geneva, Switzerland) from STMicroelectronics was selected as the data processing chip, using an external quartz crystal oscillator as the clock source. A low-ripple linear voltage regulator chip, AMS1117 (MSKSEMI, Taiwan, China), converts 5 V to 3.3 V and is used to power the microcontroller. The STM32F401CBU6 is a 32-bit microcontroller based on the Cortex-M4 core, with a maximum clock frequency of 84 MHz. It features a built-in FPU, supports various peripherals, and is housed in a compact UFQFPN48 package. The main characteristics are as follows:

- A wide input voltage range of 1.7 V to 3.6 V.
- A built-in 256 KB Flash and 64 KB SRAM, providing ample storage and programming space.
- A current consumption of 128 μA/MHz in run mode and 42 μA in sleep mode, ensuring a very low power consumption.
- A 16-channel DMA with FIFO and interrupt capabilities.
- Up to 11 multifunctional timers.
- A total of 11 communication interfaces, including 3 USARTs, 3 I2Cs, and 4 SPIs.

We chose the SPI as the communication protocol between the sensor and the controller. Specifically, the sensor’s SCK, SDI, and SDO are connected to the corresponding SPI pins of the controller. The sensor’s CSB1 (accelerometer chip select) and CSB2 (gyroscope chip select) are connected to the GPIO output pins of the controller. The sensor’s INT1 (interrupt 1) and INT3 (interrupt 3) are connected to the interrupt input (GPIO) pins of the controller.

During the software development process of miniaturized autonomous inertial sensors, STM32CubeMX v6.11.1 and Keil uVision5 are used for the joint development and debugging. Keil uVision, as a mainstream integrated development environment, offers an excellent compatibility with ARM Cortex-M series processors and provides a comprehensive debugging toolchain, significantly shortening the development cycle. On the other hand, CubeMX enables the graphical interaction for the chip pin function mapping, clock tree configuration, and peripheral parameter settings. Its built-in intelligent conflict detection algorithm can verify the rationality of the hardware resource configuration in real-time. After completing the hardware configuration, CubeMX can automatically generate a high-quality initialization code, greatly simplifying the development process. The combination of both tools not only ensures the reliability and maintainability of the code but also significantly improves the overall development efficiency.

## 3. Analysis of Tilt Angle and Deflection in Cantilever Beam Vibration

**Figure 2:**
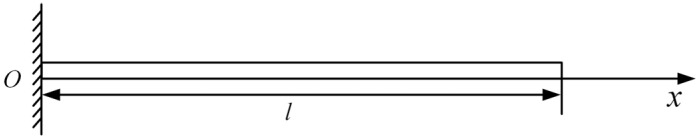
Simplified cantilever beam model.

Measuring the tilt angle in a vibrating environment is quite challenging, whereas measuring the deflection at a point is relatively simple. The tilt angle of the beam is closely related to the deflection. The relationship between the tilt angle and the deflection is analyzed below. As shown in Figure 2, a simplified cantilever beam with a constant cross-section is considered, with the length *l*, density *ρ*, cross-sectional area *A*, and flexural rigidity *EI*. Taking the left end of the cantilever beam as the origin of the coordinate system, the *x*-axis is directed along the beam to the right.

In general, the deflection of a beam is much smaller than its span, the section tilt angle *θ* is very small, and the deflection curve is relatively flat. Based on the principles of material mechanics, the approximate differential equation of the deflection curve can be derived as follows:

(1) $$\frac{d^{2}w}{dx^{2}}=\frac{M}{EI},$$

where *w* is the deflection of the cantilever beam, and *M* is the bending moment applied to the beam.

Integrating Equation (1) produces:

(2) $$\begin{array}{c} \theta =\frac{dw}{dx}=\int \frac{M}{EI}dx+C,\\ w=\iint \left(\frac{M}{EI}dx\right)dx+Cx+D, \end{array}$$

where *C* and *D* are integration constants determined by boundary conditions.

Under known bending moment conditions, the tilt angle and deflection at any point on the beam can be calculated, thereby establishing the relationship between the tilt angle and deflection.

Under small deformation assumptions, the bending deformation of the beam can be decomposed into the superposition of deformations caused by different load conditions. The vibration of the cantilever beam can be decomposed into static deformation due to gravity and the dynamic deformation unaffected by gravity during the vibration. Gravity can be modeled as a distributed load *q*. According to Equation (2), the deflection and tilt angle under gravity are given by

(3) $$\begin{array}{c} w_{g}(x)=-\frac{qx^{2}}{24EI}\left(x^{2}-4lx+6l^{2}\right),\\ \theta_{g}(x)=-\frac{q}{6EI}\left(x^{3}-3lx^{2}+3l^{2}x\right),\\ q=\rho A, \end{array}$$

**Figure 3:**
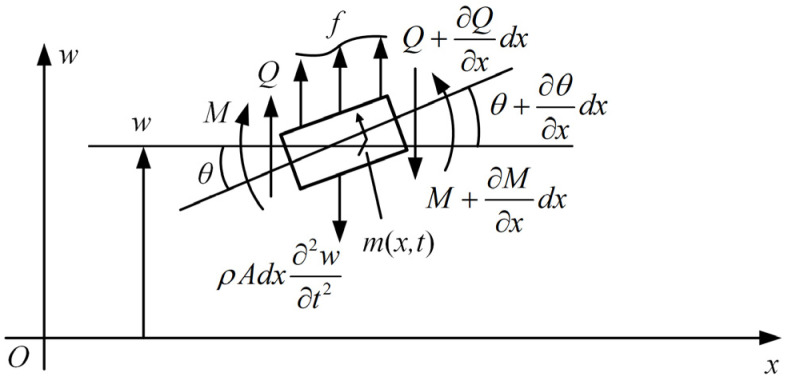
The force analysis of an infinitesimal segment of the Euler–Bernoulli beam.

The dynamic deformation during the vibration without external force conditions will be analyzed below. The previously established model can be regarded as an Euler–Bernoulli beam, with *w_f_* (*x*,*t*) representing the deflection of the neutral axis of the cross-section at position *x* and time *t*. A force analysis is conducted on a differential segment of the length *dx* of the beam, as shown in Figure 3. *Q* and *M* represent the shear force and bending moment on the left section, while *f*, *m*, and $\rho Adx\frac{\partial^{2}w}{\partial t^{2}}$ represent the external force, external moment, and inertial force acting on the differential segment of the beam, respectively.

In the absence of external forces (i.e., *f* = 0 and *m* = 0), according to Newton’s second law, we have

(4) $$\rho Adx\frac{\partial^{2}w}{\partial t^{2}}+\frac{\partial Q}{\partial x}dx=0,$$

Performing a force analysis on the right section and neglecting higher-order small terms, we obtain

(5) $$M+Qdx=M+\frac{\partial M}{\partial x}dx,$$

Substituting Equations (1) and (5) into Equation (4), the dynamic equation of motion for the free bending vibration of a homogeneous beam with a constant cross-section can be derived:

(6) $$\rho A\frac{\partial^{2}w}{\partial t^{2}}+EI\frac{\partial^{4}w}{\partial x^{4}}=0,$$

Equation (6) can be solved using the method of the separation of variables. Let its solution be

(7) w(x,t)=W(x)q(t),

where *W*(*x*) is the natural mode shape function, and *q*(*t*) is the time-dependent function.

Substituting Equation (7) into Equation (6), we obtain

(8) $$\begin{array}{c} \begin{array}{c} W(x)=a_{1}\cos(\kappa x)+a_{2}\sin(\kappa x)+a_{3}\cosh(\kappa x)+a_{4}\sinh(\kappa x),\\ q(t)=b_{1}\cos(\omega t)+b_{2}\sin(\omega t), \end{array}\\ \kappa^{4}=\frac{\rho A}{EI}\omega^{2}, \end{array}$$

where κ is the wavenumber of the natural mode shape function *W*(*x*) along the length of the beam, and *ω* is the natural frequency.

Equation (8) describes the distribution of the bending vibration amplitude along the beam and the time-dependent function of the beam’s vibration. The parameters *ω*, *a*_1_, *a*_2_, *a*_3_, *a*_4_, *b*_1_, and *b*_2_ are determined by the boundary and initial conditions.

For a cantilever beam, the left end is fixed, with boundary conditions of *w* (0) = 0 and *θ*(0) = 0. The right end is a free boundary, with boundary conditions of *M* (*l*) = 0 and *Q* (*l*) = 0. Substituting these boundary conditions into Equation (8) yields the solutions:

(9) $$\kappa_{1}l=1.8751,\;\kappa_{2}l=4.6941,\;\kappa_{3}l=7.8548,\;\kappa_{n}l=\frac{(2r-1)\pi }{2},n\geq 4,$$

where subscript *n* denotes the order of normal modes.

By setting *a*_1_ = 1, the normal mode shape function of the cantilever beam is obtained:

(10) $$W_{n}(x)=\cosh\left(\kappa_{n}x\right)-\cos\left(\kappa_{n}x\right)-\frac{\sinh(\kappa_{n}l)-\sin(\kappa_{n}l)}{\cosh(\kappa_{n}l)+\cos(\kappa_{n}l)}\left[\sinh\left(\kappa_{n}x\right)-\sin\left(\kappa_{n}x\right)\right],$$

Using the modal superposition method, the deflection function of the vibrating cantilever beam is expressed as

(11) $$w_{f}(x,t)=\sum_{n=1}^{+\infty }W_{n}(x)q_{n}(t),$$

The parameters of *q_n_*(*t*) are determined based on the initial state of the beam, assuming that the beam is initially subjected to a force *F* at its free end, causing it to bend, and then released from a stationary state for free vibration. In this case, the beam’s deflection equation is

(12) $$w_{f}(x,0)=-\frac{Fx^{2}}{6EI}(3l-x),$$

According to the orthogonality of normal modes

(13) $$\begin{array}{c} M_{n}{\ddot{q}}_{n}(t)+K_{n}q_{n}(t)=f_{n}(t),\\ q_{n}(0)=\frac{1}{M_{n}}\int_{0}^{l}\rho Aw_{0}(x)W_{n}(x)dx,\\ {\dot{q}}_{n}(0)=\frac{1}{M_{n}}\int_{0}^{l}\rho Av_{0}(x)W_{n}(x)dx, \end{array}$$

where *M_n_*, *K_n_*, and *f_n_* represent the *n*th order modal mass, modal stiffness, and modal force, respectively. *w*_0_(*x*) is the deflection curve of the beam at the initial moment, and *v*_0_(*x*) is the velocity of the beam at the initial moment.

The vibration equation of the cantilever beam is solved as

(14) $$w_{f}(x,t)=\sum_{n=1}^{+\infty }W_{n}(x)\left[q_{n}(0)\cos\omega_{n}t+\frac{{\dot{q}}_{n}(0)}{\omega_{n}}\sin\omega_{n}t\right],$$

The free bending vibration of the beam primarily consists of the first-order natural vibration mode, and the contribution from higher-order modes can be ignored. Moreover, ${\dot{q}}_{1}(0)$ = 0, thus

(15) $$w_{f}(x,t)=W_{1}(x)[q_{1}(0)\cos(\omega_{1}t)],$$

Taking the partial derivative of the deflection with respect to *x* yields the function for the section’s tilt angle:

(16) $$\theta_{f}(x,t)=\frac{\partial w_{f}(x,t)}{\partial x}={\dot{W}}_{1}(x)\left[q_{1}(0)\cos\omega_{1}t\right],$$

From Equations (15) and (16), it can be seen that there is a proportional relationship between the deflection and tilt angle at a certain location. The tilt angle on a cantilever beam is difficult to determine, while the deflection measurement is relatively easier. Therefore, to measure the tilt angle, it is sufficient to measure the deflection at that location.

Based on the above analysis, the deflection and tilt angle during the vibration of a cantilever beam consist of two components: the static deformation due to the effect of gravity and the dynamic deformation during vibration, which is unaffected by gravity. The total deflection and total tilt angle during the vibration of the cantilever beam are

(17) $$\begin{array}{c} \theta (x,t)=\theta_{g}(x)+\theta_{f}(x,t),\\ w(x,t)=w_{g}(x)+w_{f}(x,t). \end{array}$$

## 4. Improved Enhanced Kalman Filter

In the field of inertial measurements, accelerometers and gyroscopes serve as core sensors with complementary characteristics. An accelerometer contains mutually orthogonal measurement axes. Based on the principle of gravity vector decomposition, the tilt angle is estimated by measuring the acceleration components along the orthogonal axes. Gyroscopes measure the angular velocity during the object rotation, and the tilt angle can be obtained by integrating over time. The tilt angle measured by an accelerometer is highly accurate, but it is susceptible to the interference from vibrations and acceleration in dynamic environments. Gyroscopes provide stable angular velocity measurements over short periods but are prone to zero-bias drift. By using the KF to fuse the data from both sensors, the accuracy of the tilt angle measurement can be improved. As a recursive algorithm, the KF processes and updates sensor data in real-time without storing historical data, reducing hardware requirements and enhancing the dynamic adaptability and long-term stability of the measurement system. To use a KF, a system model must first be established. The model for the KF is as follows:

The KF predicts the state at time *t* using the previous state *x_k_*_−1_ and control input *u_k_*:

(18) $$x_{k}=F(x_{k-1},k)+B\cdot u_{k}+w_{k},$$

where *x_k_* is the state at time *k*; *F* is the nonlinear state transition function; *B* is the control input matrix; and *w_k_* is the process noise.

The state observation equation is as follows:

(19) $$z_{k}=H(x_{k},k)+v_{k},$$

where *z* is the system’s observation vector; *z_k_* represents the state vector at time *k*; *H* is a nonlinear function that maps the state to the observation, describing the relationship between the system’s state and the observed values; and *v_k_* is the observation noise, representing measurement errors or uncertainties.

First, it is critical to select appropriate observation quantities for the KF. Since accelerometers are susceptible to interference in a vibrating environment, directly using their tilt angle measurements as observation inputs would adversely affect the KF’s state estimation. Firstly, large observation errors can reduce the accuracy of the state correction, leading to divergent estimation results. Secondly, the KF may overfit the noisy measurements, deviating from the system’s dynamic model and generating biased state estimates. These issues significantly affect the filter’s convergence and stability. To reduce the interference caused by the vibration, it is necessary to analyze and process the measured acceleration values and find suitable observation variables and observation equations to improve the accuracy of the tilt angle measurements.

**Figure 4:**
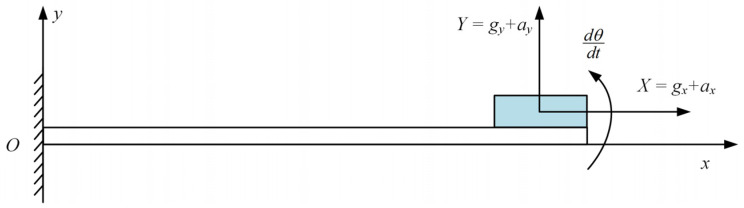
Force analysis of sensors.

The following is an analysis of the sensor under vibrating conditions, as shown in Figure 4. Where *X* and *Y* are the *x-* and *y*-axis acceleration values measured by the accelerometer, *a_x_* and *a_y_* represent the accelerations caused by motion, *g_x_* and *g_y_* represent the accelerations due to gravity, and $\frac{d\theta }{dt}$ is the angular velocity of the end face’s rotation.

From Section 3, the deflection at the measurement point is proportional to the tilt angle, i.e.,

(20) w=kθ,

Taking the second derivative of both sides with respect to time yields

(21) $$a_{y}=k\ddot{\theta },$$

Approximating the motion of the end face as circular motion, we obtain

(22) $$a_{x}=-{\left(\dot{\theta }\right)}^{2}r,$$

where *r* is the radius of the circular motion.

The acceleration measured by the accelerometer is a combination of the actual acceleration of the object and the acceleration due to gravity. Therefore,

(23) $$\begin{array}{l} X=a_{x}+g_{x}=g\sin(\theta )-(\dot{\theta }{)}^{2}r,\\ Y=a_{y}+g_{y}=g\cos(\theta )+k\ddot{\theta }, \end{array}$$

After the above analysis, the relationship between the acceleration and motion state has been established, and equations between the state and observation have been derived. Based on sensor conditions, the *x*- and *y*-axis accelerations and angular velocity are chosen as observed quantities. The observation vector *z* is

(24) $$z=\left[\begin{array}{c} Y\\ X\\ \dot{\theta } \end{array}\right],$$

According to the model, the tilt angle, angular velocity, angular acceleration, and circular radius are chosen as state variables. The state vector *x* is

(25) $$x=\left[\begin{array}{c} \theta \\ \dot{\theta }\\ \ddot{\theta }\\ r \end{array}\right],$$

From the interrelationships between the angle, angular velocity, and angular acceleration, the states between two consecutive time instants exhibit linear relationships. The nonlinear state transition function *F* is thus converted into a state transition matrix *F_k_*.

(26) $$F_{k}=\left[\begin{array}{cccc} 1 & \Delta t & 0.5\times \Delta t^{2} & 0\\ 0 & 1 & \Delta t & 0\\ 0 & 0 & 1 & 0\\ 0 & 0 & 0 & 1 \end{array}\right],$$

For the nonlinear function *H*(*x_k_*, *k*) from the state to the observation, the Jacobian matrix of the nonlinear function *H*(*x*) is denoted as *H_k_*.

(27) $${\left.\frac{\partial H}{\partial x^{T}}\right|}_{\dot{x}(k/k-1)}=\left[\begin{array}{cccc} \cos\theta & -2\times \dot{\theta }\times r & 0 & -{\dot{\theta }}^{2}\\ -\sin\theta & 0 & k & 0\\ 0 & 1 & 0 & 0 \end{array}\right]=H_{k},$$

The EKF process operates in two primary phases: prediction and updating. The state prediction equation represents the prior estimate of the state at time *k* derived from the state at time *k* − 1; the state update process uses measurement values to update the prior estimate.

The state prediction process is as follows:

(28) $$\begin{array}{c} {\hat{x}}_{k}^{-}=F_{k}\cdot {\hat{x}}_{k-1},\\ P_{k}^{-}=F_{k}\cdot P_{k-1}\cdot F_{k}^{T}+Q_{k}, \end{array}$$

where ${\hat{x}}_{k}^{-}$ is the prior prediction value obtained from the previous moment’s state; ${\hat{x}}_{k-1}$ is the posterior state value from the previous moment; $P_{k}^{-}$ is the prior covariance matrix obtained from the previous moment’s state; *P_k_*_− 1_ is the posterior covariance matrix from the previous moment; and *Q_k_* is the process noise covariance matrix, representing the uncertainty in the state prediction process.

The state update process is as follows:

(29) $$\begin{array}{c} K_{k}=P_{k}^{-}\cdot H_{k}^{T}\cdot {\left(H_{k}\cdot P_{k}^{-}\cdot H_{k}^{T}+R_{k}\right)}^{-1},\\ {\hat{x}}_{k}={\hat{x}}_{k}^{-}+K_{k}\cdot \left(z-H\left({\hat{x}}_{k}^{-},k\right)\right),\\ P_{k}=\left(I-K_{k}\cdot H_{k}\right)\cdot P_{k}^{-}. \end{array}$$

where *K_k_* is the Kalman gain; *R_k_* is the observation noise covariance matrix; and ${\hat{x}}_{k}$ is the predicted state estimate.

This iterative procedure enables the EKF to perpetually enhance the system’s state estimation by utilizing new information.

## 5. Simulation and Experiment

This section uses MATLAB 2023b and COMSOL Multiphysics® 6.3 to perform numerical and finite element simulations of the first-order bending vibration of a cantilever beam. A sensor performance verification platform is also established to validate the measurement accuracy and real-time capability of the proposed vibration sensing method.

### 5.1. MATLAB Simulation and Analysis

A dynamic model for the free vibration of the cantilever beam was established in MATLAB. Based on experimental data, the simulation parameters were configured as follows: vibration frequency *f* = 3.08 Hz, *k* = 0.683, *r* = 0.932 m, and damping ratio *ξ* = 0.0063.

**Figure 5:**
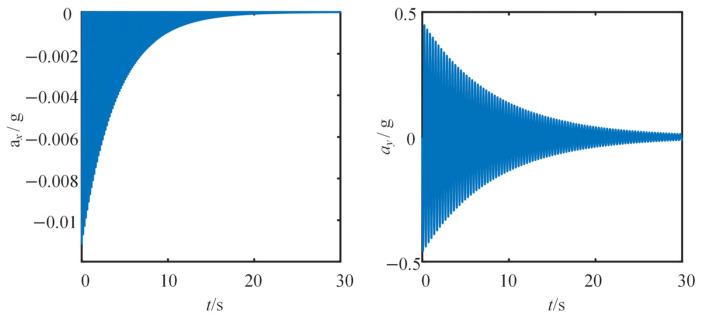
Time domain curve of *x*-axis acceleration *a_x_* and *y*-axis acceleration *a_y_*.

**Figure 6:**
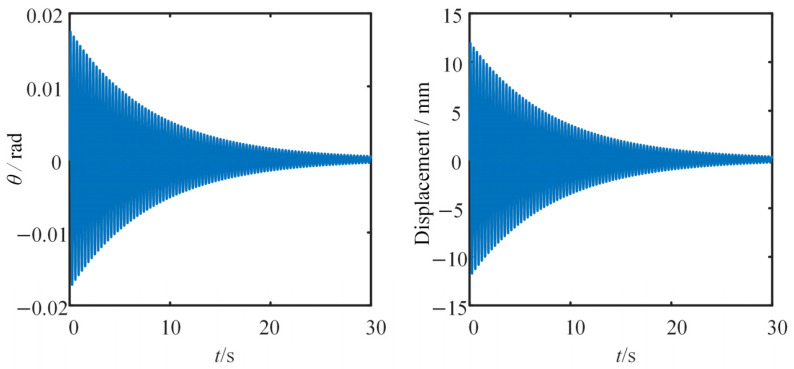
The time domain curve of the tilt angle *θ* and displacement at the end of the beam.

Figure 5 shows the time domain curve of the *x*-axis acceleration *a_x_* and *y*-axis acceleration *a_y_*, respectively. Figure 6 shows the time domain variations in the tilt angle and the displacement at the free end of the beam.

Under the effect of the damping, the vibration of the beam slowly decays. It can be observed that, after 30 s, the accelerations *a_x_* and *a_y_* caused by the motion almost reduce to zero. Correspondingly, the beam essentially stops vibrating, with both the tilt angle and displacement at the free end stabilizing. The final tilt angle and displacement converge to zero.

### 5.2. COMSOL Simulation and Analysis

The model was constructed based on COMSOL Multiphysics. Geometrically, two interconnected rectangular blocks were used to establish the cantilever beam and sensor structure. We selected the Solid Mechanics physical field and applied a fixed constraint to one end of the beam. A structured swept mesh was employed, and a mesh independence verification was performed. The modal analysis results show that the system’s first natural frequency is 3.11 Hz, which deviates less than 1% from the experimentally measured value of 3.08 Hz, validating the effectiveness of the model.

**Figure 7:**
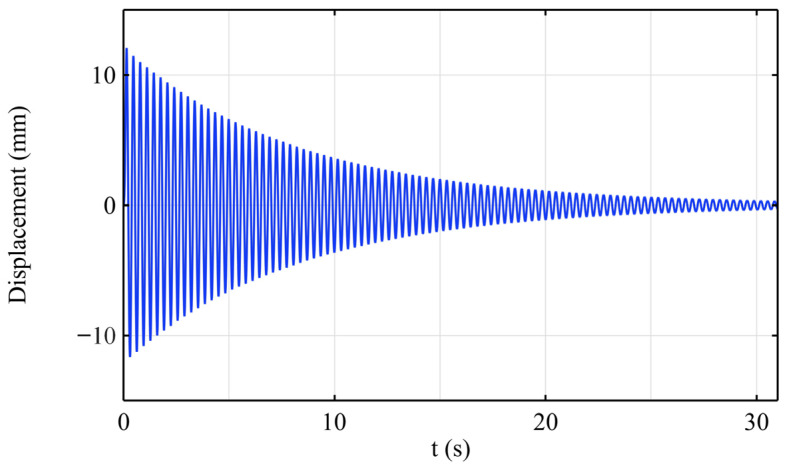
The free vibration displacement attenuation at the free end of the cantilever beam.

**Figure 8:**
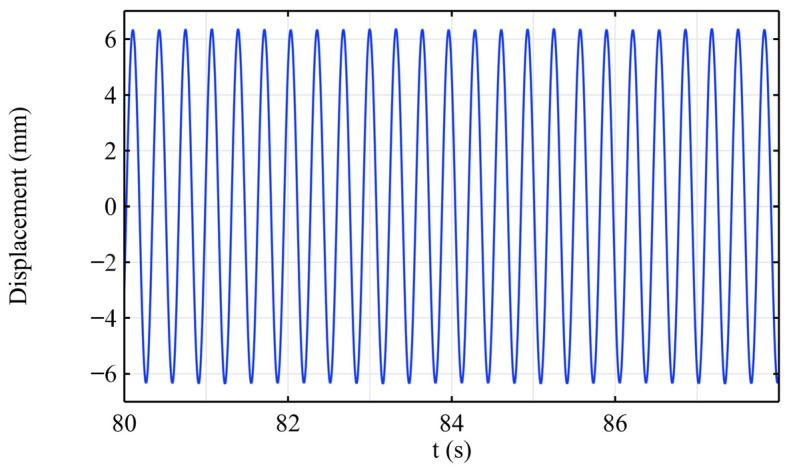
The steady-state vibration displacement at the free end of the cantilever beam.

In the transient analysis, a 0.1 s pulse load was first applied at the free end. The time-dependent displacement curve of the cantilever beam’s free end under the free vibration decay is shown in Figure 7. It can be observed that the structural vibration almost completely decays to zero within 30 s. The dynamic characteristics are consistent with both the MATLAB numerical simulation and experimental results. Further, a sinusoidal excitation load with a frequency of 3.11 Hz is applied at the free end of the beam, and the steady-state vibration response of the structure is obtained through the simulation. Figure 8 shows the time variation in the displacement at the free end after the vibration stabilizes, which is consistent with the experimental results.

### 5.3. Experiment and Analysis

**Figure 9:**
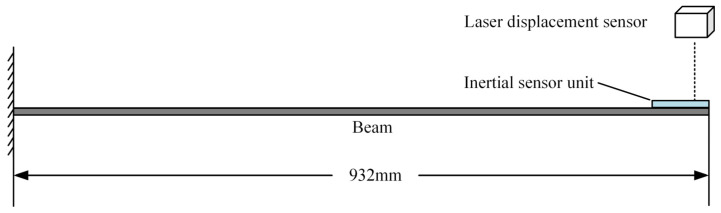
Sensor performance verification platform.

To validate the performance of the developed inertial sensor, an experimental platform based on a cantilever beam structure was built, as shown in Figure 9. The cantilever beam was made of 45 steel, with a length of 932 mm, a width of 40 mm, and a thickness of 4 mm. The inertial sensor was mounted at the free end of the cantilever beam. At the same time, the high-performance laser Doppler vibrometer OFV-505/5000 (Polytec GmbH, Waldbronn, Germany) is used for high-precision reference displacement measurements for the comparative analysis. Its displacement resolution is better than 0.05 pm, and the frequency range is from 0 to 2.5 MHz. The IMU’s cut-off frequency was set to 80 Hz, with a data output frequency of 800 Hz. This configuration effectively filters out high-frequency noise while preserving valid data, thereby enhancing the measurement accuracy and reducing interference. Before the experiment began, we also performed zero-bias calibration on the IMU based on the horizontal reference plane.

**Figure 10:**
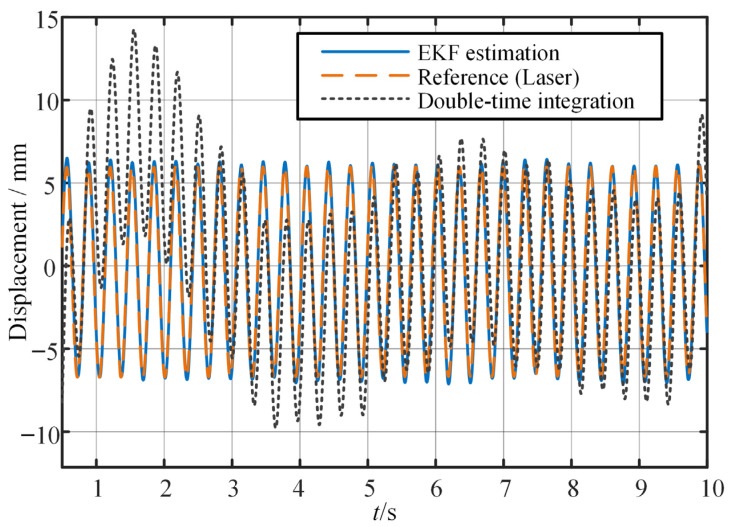
Time domain displacement measured by two sensors under steady-state vibration.

First, a modal analysis is performed to determine the system’s first-order natural frequency, which is 3.08 Hz. Subsequently, a synchronous sinusoidal excitation load is applied to obtain the steady-state vibration response of the structure. As shown in Figure 10, the blue solid line and the orange dashed line represent the displacement variation curves at the free end of the beam measured by the inertial sensor and the laser displacement sensor, respectively. It can be seen that the trends of the two curves match closely, with the maximum amplitude deviation within 4%. We also calculated the displacement from the measured acceleration signal using dual time integration and subsequent high-pass filtering with a cut-off frequency of 0.2 Hz and added the displacement to the graph as shown by the black dotted line. The results indicate that the displacement prediction using the enhanced EKF yields better results.

**Figure 11:**
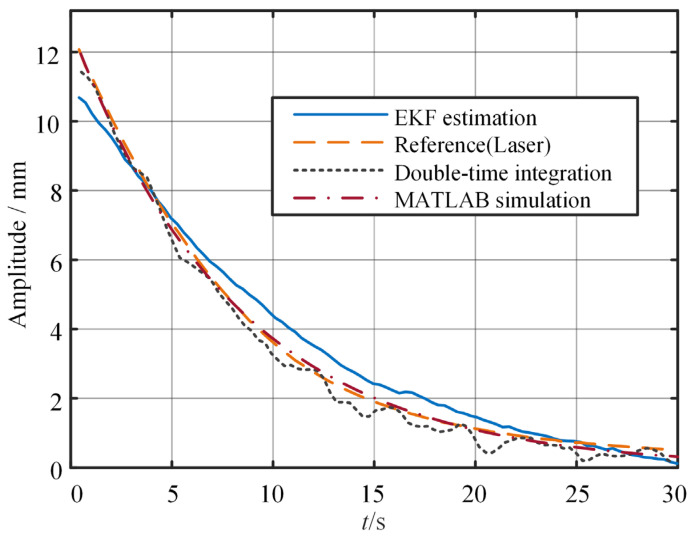
Time domain amplitude under free vibration.

**Figure 12:**
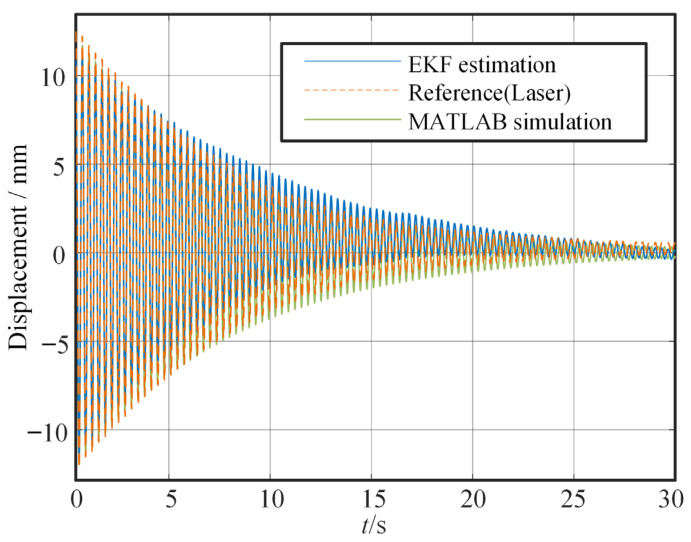
Time domain displacement under free vibration.

In the free vibration test, the cantilever beam, pre-lifted and then released, is used to assess the sensor performance throughout the entire attenuation process. Figure 11 and Figure 12 show the time domain curves of the amplitude and displacement at the end of the beam under the free vibration, respectively. Figure 11 shows that under free vibration conditions, the amplitude deviation is large initially (approximately 11%), but it decreases to a lower level over time. Notably, the vibration attenuation trend and phase characteristics output by both sensors are identical, confirming the inertial sensor’s effective capture of the structural bending vibration and demonstrating its reliability in providing critical dynamic information for vibration control. Compared with the results of the double-time integration, the results obtained by the EKF are also more stable. Furthermore, a comparison between experimental and simulation results also reveals small differences.

## 6. Conclusions

This paper develops a miniaturized autonomous inertial sensor aimed at overcoming the challenges of integrating vibration sensors into active or semi-active vibration control systems, particularly in structures that are distant from the main body. The sensor primarily incorporates a BMI088 IMU and an STM32F401CBU6 microcontroller unit for data processing. This standalone design provides better shock resistance. It can be easily attached to the controlled structure to measure vibration information and enable wireless data transmission. The relationship between the vibration characteristics of the cantilever beam structure and the inertial acceleration and angular velocity measured by the sensor is established. In addition, an improved EKF algorithm is employed in the algorithm design to correct the state variables of the accelerometer, effectively eliminating the dynamic interference in the vibration environment and enhancing the real-time data processing capability and anti-interference performance of the sensor. Through simulations and experiments, it is demonstrated that the inertial sensor exhibits an engineering-grade accuracy in extracting structural bending vibration characteristics. Its dynamic response, consistency, and low-latency characteristics provide a lightweight solution for real-time vibration monitoring.

## Figures and Tables

**Table 1:** Specific parameters of the BMI088.

	Accelerometer	Gyroscope
Range	24 g	2000°/s
Temperature drift	0.002%/K (max)	0.03%/K (max)
Zero drift	20 mg (max)	1°/s (max)
Cut-off frequency	280 Hz (max)	230 Hz (max)
Noise	$190\;\mathrm{mg}/\sqrt{\mathrm{Hz}}$	0.1%/K
Data rate	1600 Hz (max)	2000 Hz (max)

## Data Availability

The data is contained within the article.

## Abbreviations

The following abbreviations are used in this manuscript:

IMU	Inertial Measurement Unit
KF	Kalman Filter
EKF	Extended Kalman Filter
MEMSs	Micro-Electro-Mechanical Systems
GPS	Global Positioning System
GNSS	Global Navigation Satellite System
RMS	Root Mean Square
FPC	Flexible Printed Circuit
SPI	Serial Peripheral Interface
PCB	Printed Circuit Board
FPU	Floating-Point Unit
